# Incidental findings on coronary computed tomography in women with selected reproductive disorders

**DOI:** 10.1186/s13244-022-01238-z

**Published:** 2022-06-04

**Authors:** Kim van der Ham, Charissa van Zwol-Janssens, Birgitta K. Velthuis, Maria P. H. Koster, Yvonne V. Louwers, Dustin Goei, Maurits S. H. Blomjous, Arie Franx, Bart C. J. M. Fauser, Eric Boersma, Joop S. E. Laven, Ricardo P. J. Budde, the CREW Consortium

**Affiliations:** 1 Division of Reproductive Endocrinology and Infertility, Department of Obstetrics and Gynaecology, Erasmus University Medical Center, Dr. Molewaterplein 40, 3015 GD Rotterdam, The Netherlands; 2 Department of Radiology, University Medical Center Utrecht, University of Utrecht, Heidelberglaan 100, 3584 CX Utrecht, The Netherlands; 3 Department of Obstetrics and Gynaecology, Erasmus University Medical Center, Dr. Molewaterplein 40, 3015 GD Rotterdam, the Netherlands; 4 Department of Reproductive Medicine and Gynaecology, Department of Obstetrics and Gynaecology, University Medical Center Utrecht, University of Utrecht, Heidelberglaan 100, 3584 CX Utrecht, The Netherlands; 5 Department of Cardiology, Erasmus University Medical Center, Dr. Molewaterplein 40, 3015 GD Rotterdam, the Netherlands; 6 Department of Radiology and Nuclear Medicine, Erasmus University Medical Center, Dr. Molewaterplein 40, 3015 GD Rotterdam, the Netherlands

**Keywords:** Polycystic ovary syndrome, Premature ovarian insufficiency, Preeclampsia, Incidental findings, Computed tomography

## Abstract

**Objective:**

To determine the prevalence of incidental findings (IFs) on coronary computed tomography (CCT) in women aged 45–55 years and previously diagnosed with reproductive disorders such as polycystic ovary syndrome (PCOS), premature ovarian insufficiency (POI) or preeclampsia (PE).

**Methods:**

A total of 486 middle-aged women with PCOS (*n* = 101), POI (*n* = 97) or a history of PE (*n* = 288) underwent a CCT as part of a prior prospective study. IFs were categorized by their significance (minor, moderate and major). Follow-up information was collected from patients’ records. To investigate the impact of different field of views (FOVs), a subset of scans was analyzed in full FOV and small FOV.

**Results:**

In 96/486 (19.8%) women, one or more IFs were detected, of which 54/486 (11.1%) were classified as moderate/major and 48/486 (9.9%) required follow-up. A moderate/major IF was detected in 16/101 (15.9%) women with PCOS, 13/97 (13.4%) women with POI and 25/288 (8.7%) women with a history of PE. In 78 women with an IF detected in the full FOV, the IF was still visible in 60 (76.9%) women in the small FOV. In the full FOV, 46 women required follow-up, but using the small FOV this was reduced to 30 women.

**Conclusion:**

Using CCT as a cardiovascular disease screening tool in women with selected reproductive disorders increases the probability of detecting IFs that can cause anxiety and may generate extra costs, but can also reveal clinically relevant findings. Using a small FOV centered around the heart resulted in a lower prevalence of IFs and required less follow-up.

## Key points

- Using CT scans as a cardiovascular screening tool increases the probability of detecting incidental findings in middle-aged women with reproductive disorders.
- Incidental findings can potentially cause anxiety and may generate extra costs for downstream diagnostic testing but can also reveal clinically relevant findings.
- Using a reconstructed small field of view resulted in a lower prevalence of incidental findings and required less follow-up diagnostics.

## Introduction

Women with reproductive disorders, including polycystic ovary syndrome (PCOS), premature ovarian insufficiency (POI) and hypertensive pregnancy disorders (HPD), such as preeclampsia (PE), are more prone to develop cardiovascular diseases (CVD) later in life [1–4]. For example, PCOS is associated with cardiometabolic risk factors, such as obesity, dyslipidemia, type II diabetes, hypertension and metabolic syndrome, all of which increase the risk of CVD [5, 6]. Women with POI exhibit an unfavorable cardiovascular risk profile, including higher abdominal fat, elevated chronic inflammatory factors and a predisposition toward hypertension [7]. Women experiencing PE in pregnancy have an increased risk of developing hypertension and subclinical atherosclerosis at age 45–55 years [8]. This increased risk of CVD in women with reproductive disorders has many underlying mechanisms and highlights the importance of cardiovascular screening of this population [9].

Coronary computed tomography (CCT) can be used as a screening tool to detect presymptomatic coronary artery disease (CAD). However, this is not standard practice for women with reproductive disorders. CCT opens the possibility of recognizing and treating milder forms of CAD with lifestyle recommendations and/or medical therapy to prevent serious CVD later on. However, CCT also introduces the problem of detecting incidental findings (IFs). An IF is an unexpected new finding on any medical test that is not the aim of the test and may have potential health consequences [10, 11]. The prevalence of IFs in research chest imaging is around 30%, depending on the study population [12] and can be more prevalent than the target of the scan. Visualizing and reporting IFs is a matter of debate. Although some IFs can reveal a treatable disease or cancer in an early stage, many IFs are irrelevant and often lead to more downstream diagnostic testing, resulting in higher healthcare costs and increased anxiety for the patient [13, 14].

No previous studies have investigated the prevalence of IFs in middle-aged women with reproductive disorders. In this study, we provide a descriptive analysis of the prevalence of IFs in women aged 45–55 years with PCOS, POI or a history of PE who underwent CCT as part of a previous published study, to assess cardiovascular disease in these women [15].

## Materials and methods

### Study design and study setting

In this cross-sectional study, we investigated the prevalence and nature of IFs in women with PCOS, POI or a history of PE that underwent CCT as part of the CREw-IMAGO study [15]. The rationale and design of the CREw-IMAGO study (Cardiovascular Risk Profile: Imaging and Gender-Specific Disorders) have been published previously (URL: http://www.trialregister.nl/trialreg/index.asp. Unique identifier: NTR5531) [15]. In short, the aim of this study was to assess CVD by using CCT in patients with a history of selected reproductive disorders. The study was performed at two University Medical Centers, in Utrecht (UMC Utrecht) and Rotterdam (Erasmus MC), in the Netherlands and approved by the Medical Research Ethics Committee (METC number 15-508). All participants provided written informed consent.

### Study participants

All patients with one of the three mentioned reproductive disorders underwent regular cardiovascular screening at a specialized vascular outpatient clinic in the participating hospitals, as part of standard preventive care. The study population was composed of women aged 45–55 years who were diagnosed during their reproductive age with one of the following disorders:

1. PCOS, as defined by Rotterdam consensus criteria [16], requiring the presence of at least two of the following criteria: (1) oligo − /anovulation, (2) clinical and/or biochemical hyperandrogenism and (3) polycystic ovaries on ultrasonography.

2. POI, as defined in the Dutch Society of Obstetrics and Gynaecology (NVOG) guideline [17], requiring the presence of a secondary amenorrhea for at least 4 months accompanied by elevated FSH levels above 40 IU/L, occurring prior to the age of 40.

3. A history of PE, as defined by the International Society for the Study of Hypertension in Pregnancy criteria [18], including early-onset PE (i.e., delivery before 34 weeks of gestation) and late-onset PE (i.e., delivery after 34 weeks of gestation).

Information on ethnicity, educational level, smoking, alcohol use, oral contraceptive, or hormone replacement therapy (HRT) use, parity and menopausal status were obtained from questionnaires prior to the CCT. Age was calculated based on date of birth and date of CCT. Women’s height and weight were measured during a visit for the non-invasive vascular measurement, and body mass index (BMI, kg/m^2^) was calculated.

### Coronary CT imaging

Participants underwent non-contrast-enhanced CCT to assess the coronary calcium (CAC) score, followed by contrast-enhanced CT angiography (CCTA) to assess coronary artery atherosclerosis and stenosis. The CCT imaging protocol has been published previously [15]. Relevant CAD was defined by luminal stenosis of ≥ 50% or a CAC score ≥ 100 AU.

**Fig. 1:**
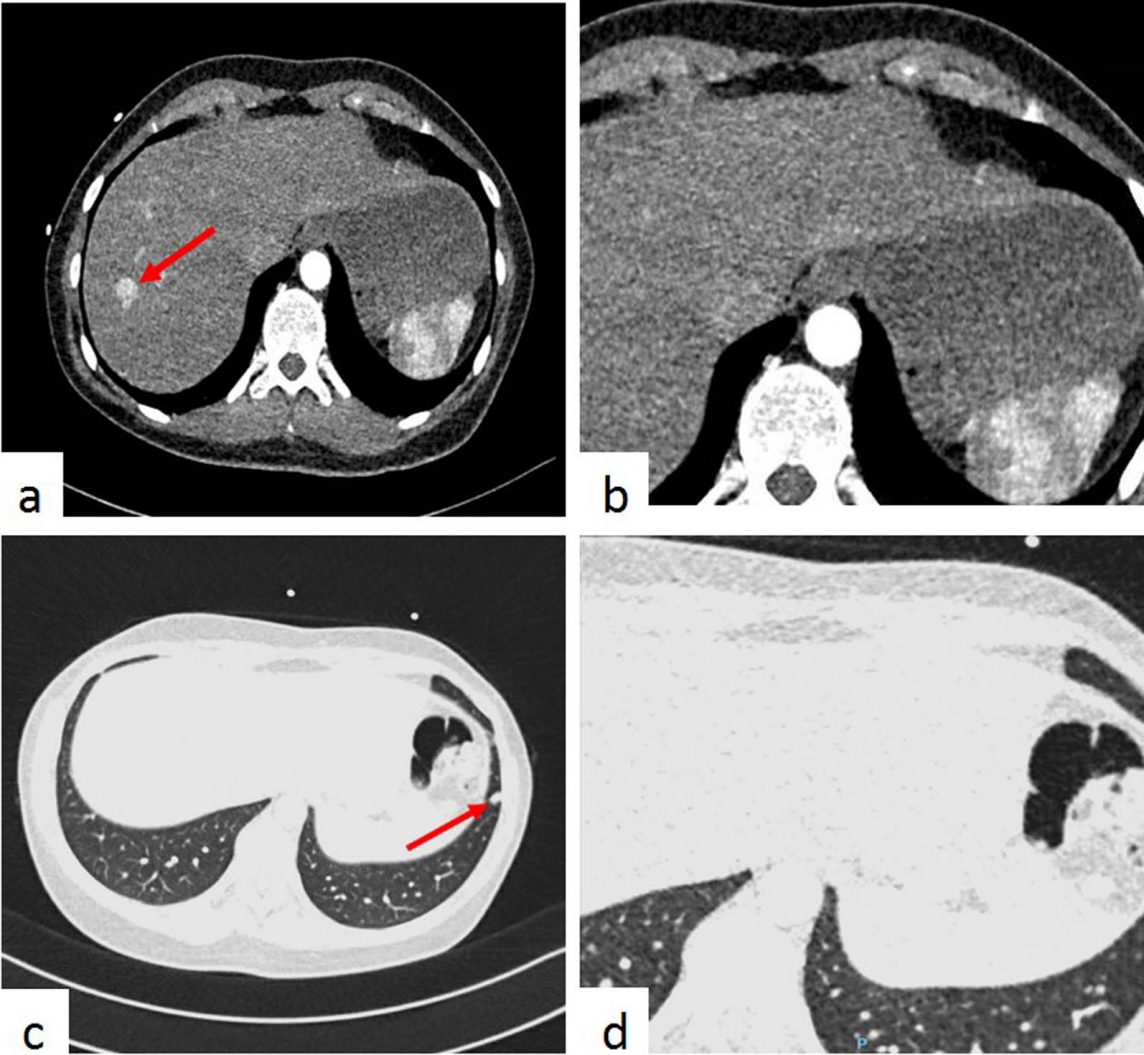
Examples of a moderate liver and a moderate lung IF in different field of views. **a** An example of a moderate liver IF (a hypervascular lesion) in a full field of view (FOV) with a ‘soft tissue’ window level (C:40 W:400). **b** The same CTA as **a**, but in a small FOV, in which the moderate liver IF falls beyond the area of this reconstruction. **c** An example of a moderate lung IF (a lung nodule) in a full FOV with a ‘lung’ window level (C:-600 W:1200). **d** The same CTA as **c**, but in the small FOV, in which the lung nodule falls beyond the area if this reconstruction

**Fig. 2:**
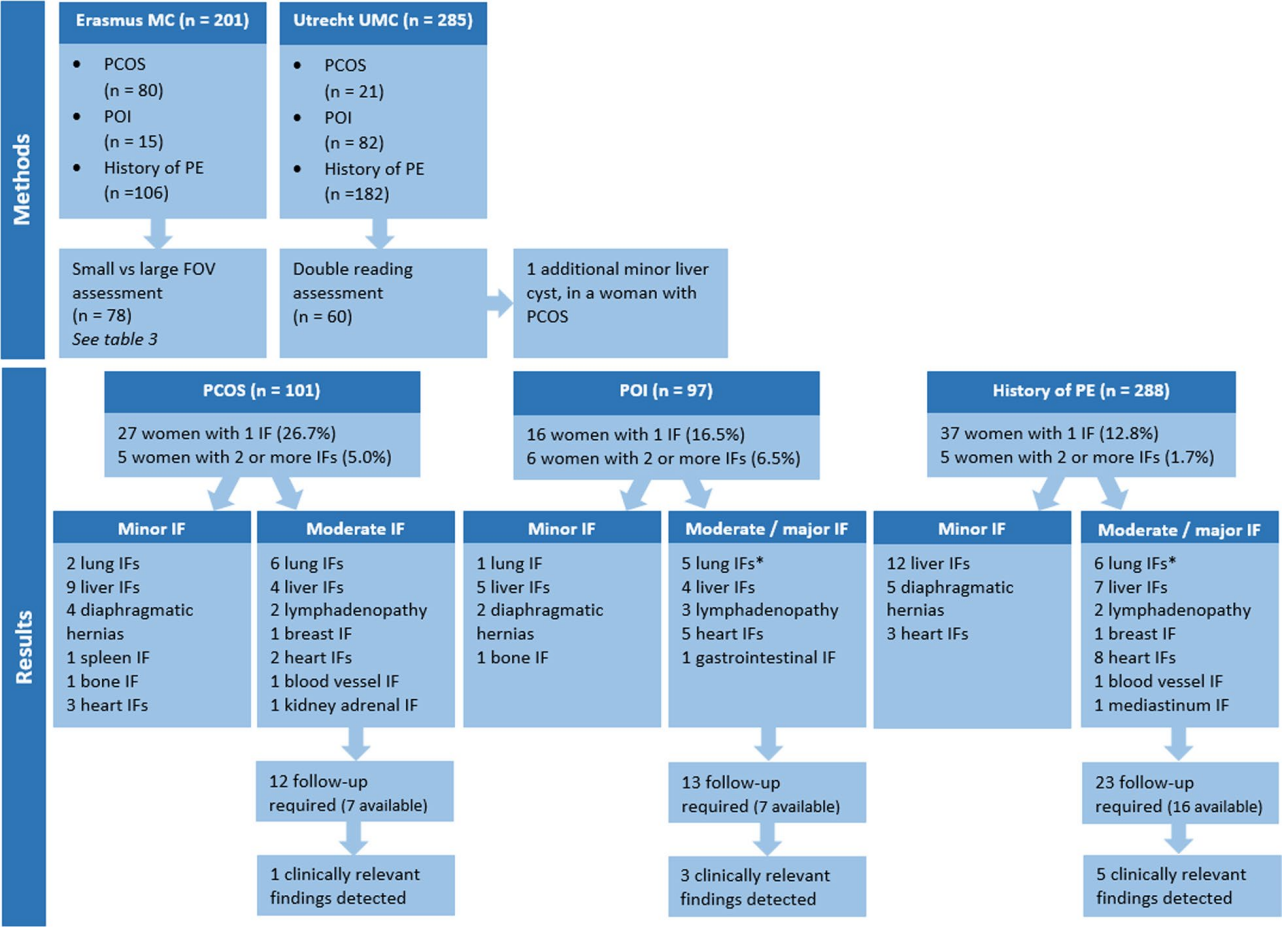
Flowchart methods and results. The IFs shown in this figure are IFs detected in the small FOV. *Major IFs, in the POI group it included 2 major lung IFs and in the PE group 1 major lung IF

All CCTs in both centers were reconstructed according to the local standard protocol. The CCTs in UMC Utrecht were made on a 256-slice scanner (iCT, Philips Healthcare, Best, the Netherlands), reconstructed with a small field of view (FOV) of 180 mm centered around the heart (Fig. 1) and assessed for CAD and IFs by a single experienced cardiovascular radiologist. All CCTs performed in Erasmus MC were made on a dual-source scanner (Somaton Force or Drive Siemens CT, Siemens, Forchheim, Germany), reconstructed in both a small FOV of 180 mm and a full FOV, from skin to skin in the irradiated area (Fig. 1), and assessed for CAD and IFs by an experienced cardiovascular imaging fellow (qualified radiologist) and a cardiovascular radiologist (so called double reading). To investigate the potential added value of this double reading, 60 (21.1%) random CCTs from the UMC Utrecht were additionally assessed for IFs by a cardiovascular imaging fellow (qualified radiologist) in this center (Fig. 2). To investigate the impact of using different FOVs, all CCTs with an IF at Erasmus MC (*n* = 78) were additionally evaluated in a smaller FOV (180 mm) to establish whether IFs were still visible (Fig. 2).

### Incidental findings

All IFs were reported by (fellow) radiologists, and the potential implications for medical management were scored by two researchers. Disagreements in scoring were resolved through consensus or by an experienced radiologist. In the result section as well as in Table 2 and Fig. 2, the IFs are shown as IFs detected in the small FOV, and in the last part of the result section IFs in both FOVs will be demonstrated.

IFs were categorized in three groups, based on the scoring system of the Royal College of Radiologists [12]:

- Major: Always requiring further investigation and likely to have adverse health effects.
- Moderate: Usually requires further investigation but health effects are unclear.
- Minor: Rarely requires further investigation and unlikely to have adverse health effects.

Information about follow-up was extracted from patient records if follow-up was performed in the same hospital as where the CCTs were performed. However, some women were referred back to their general practitioner or referral hospital for the necessary follow-up. Because of privacy regulations, we were not able to retrieve this follow-up information.

### Statistical analysis

Baseline characteristics were expressed as medians with interquartile ranges or numbers with percentages. The IFs were presented as number of women with one or more IF(s) with percentages.

## Results

### Prevalence and nature of incidental findings

We included 101 women with PCOS, 97 women with POI and 288 women with a history of PE. Table 1 shows the baseline characteristics. The median age of women with PCOS was 48.6 years (46.7–53.2), of women with POI 48.2 years (46.5–51.9) and of women with a history of PE 46.2 years (43.7–49.3). The following demonstrated IFs are IFs detected in the small FOV. In the last part of the results, the differences of both FOVs are shown. IFs were detected in 32/101 (31.7%) women with PCOS, in 22/97 (22.7%) women with POI and in 42/288 (14.6%) women with a history of PE. As part of the cardiovascular screening in the CREw IMAGO study, relevant CAD was detected in 5.4% of women with PCOS, in 3.1% of women with POI and in 6.7% of women with a history of PE.

**Table 1:** Baseline characteristics

	PCOS (*n* = 101)	POI (*n* = 97)	PE (*n* = 288)
Age, median (IQR) (years)	48.6 (46.7–53.2)	48.2 (46.5–51.9)	46.2 (43.7–49.3)
BMI, median (IQR) (kg/m^2^)	27.3 (23.6–32.0)	24.9 (22.3–27.7)	26.5 (23.4–30.4)
Ethnicity (Northern-European), *n* (%)	86 (86.9)	94 (96.9)	123 (90.4)
Educational level, *n* (%)			
Low	15 (16.0)	18 (19.4)	41 (15.0)
Middle	41 (43.6)	34 (36.6)	109 (39.8)
High	38 (40.4)	41 (44.1)	124 (45.3)
Ever smoker, *n* (%)	39 (39.4)	47 (49.5)	104 (37.7)
Current alcohol use, *n* (%)	64 (64.6)	70 (75.3)	192 (68.8)
Oral contraceptive use, *n* (%)			
Ever	77 (77.8)	90 (100.0)	242 (89.0)
Never	9 (9.1)	–	10 (3.7)
Now	13 (13.1)	–	20 (7.4)
Nulliparous, *n* (%)	17 (20.0)	10 (16.1)	–
Menopausal status, *n* (%)	27 (31.0)	97 (100.0)	99 (40.1)
HRT use, *n* (%)			
Ever	8 (27.6)	59 (67.0)	4 (4.9)
Never	21 (72.4)	27 (30.7)	73 (89.0)
Now	–	2 (2.3)	5 (6.1)
Relevant coronary artery disease^a^	5 (5.4)	3 (3.1)	18 (6.7)
Women with IF(s)	32 (31.7)	22 (22.7)	42 (14.6)

Values are observed data and represent medians (IQR) or number of patients (valid %). The presented data of IFs are minor, moderate and major IFs, all detected in the small field of view

IQR, interquartile range; PCOS, polycystic ovary syndrome; POI, primary ovarian insufficiency; PE, preeclampsia; BMI, body mass index; HRT, hormone replacement therapy; IF, incidental finding. ^a^Relevant coronary artery disease = luminal stenosis of ≥ 50% or a CAC score ≥ 100 AU

#### Women with PCOS

In the PCOS group using the small FOV, 15/101 (14.9%) women had one moderate IF on CCT, 1/101 (1.0%) women had two moderate IFs and follow-up was required in 12/101 (11.9%). These IFs were most frequently found in the lungs (*n* = 6) and liver (*n* = 4) (Fig. 2 and Table 2). Follow-up information was available for 5/6 women with lung IFs. One woman was found to have recurrent pneumonia, and she was diagnosed with impaired anti-polysaccharide antibody response and was treated accordingly. The other four women were discharged from further follow-up because the IFs were classified as irrelevant during follow-up. For 3/4 women with a moderate liver IF, follow-up information was available. One woman was already known with a multinodular liver; therefore, no follow-up diagnostics was needed. Another woman underwent an abdominal ultrasound which confirmed the IF was a hemangioma. In the third case, an abdominal radiologist was consulted who concluded that the liver lesion seen on CCT was a hemangioma and no further follow-up was required. In 20/101 (19.8%) women, one or more minor IFs were detected, the most frequently found IFs were cysts in the liver and a diaphragmatic hernia. More details on the minor IFs detected in women with PCOS are presented in Fig. 2.

**Table 2:** Moderate/major incidental findings in the three different patient populations

	PCOS (*n* = 101)	POI (*n* = 97)	PE (*n* = 288)	Total (*n* = 486)
Women with 1 IF	15 (14.9)	7 (7.2)	23 (8.0)	45 (9.3)
Women with 2 or more IFs	1 (1.0)	6 (6.2)	2 (0.7)	9 (1.9)
Lung	6 (5.9)	5 (5.2)^±^	6 (2.1)^†^	17 (3.5)
Liver	4 (4.0)	4 (4.1)	7 (2.4)	15 (3.1)
Lymphadenopathy	2 (2.0)	3 (3.1)	2 (0.7)	7 (1.4)
Breast	1 (1.0)	–	1 (0.3)	2 (0.4)
Heart	2 (2.0)	5 (5.2)	8 (2.8)	15 (3.1)
Gastrointestinal tract	–	1 (1.0)	–	1 (0.2)
Blood vessels	1 (1.0)	–	1 (0.3)	2 (0.4)
Kidney/adrenal	1 (1.0)	–	–	1 (0.2)
Mediastinum	–	–	1 (0.3)	1 (0.2)
Follow-up required	12 (11.9)	13 (13.4)	23 (8.0)	48 (9.9)
Follow-up performed in EMC or UMCU	7 (58.3*)	7 (53.8*)	16 (69.6*)	30 (62.5*)
Follow-up performed by:				
Ultrasound	1 (16.7**)	2 (28.6**)	5 (31.3**)	8 (28.6**)
CT scan or MRI	5 (83.3**)	3 (42.9**)	6 (37.5**)	14 (46.7**)
PET scan	–	–	2 (12.5**)	2 (7.1**)
X-ray	–	–	1 (6.3**)	1 (3.6**)
Consultation	1 (14.3**)	2 (28.6**)	2 (12.5**)	5 (16.7**)
Clinically relevant Findings ^a^	1 (14.3**)	3 (42.9**)	5 (31.3**)	9 (32.1**)

Values are observed data in number of women with moderate or major incidental findings (% valid). This table shows the detected IFs in the small field of view (FOV)

PCOS, polycystic ovary syndrome; POI, primary ovarian insufficiency; PE, preeclampsia; IF, Incidental finding; EMC, Erasmus Medical Center; UMCU, University Medical Center Utrecht; CT, computed tomography; MRI, magnetic resonance imaging

^±^The lung IFs of 2 women were classified as major

^†^The lung IF of one woman was classified as major

^a^Clinically relevant findings = incidental findings which required further treatment

*Percentage of follow-up required

**Percentages of follow-up performed in EMC or UMCU

#### Women with POI

In the POI group using the small FOV, 7/97 (7.2%) women had one moderate/major IF, 6/97 (6.2%) women had two or more moderate/major IFs and in 13/97 (13.4%) follow-up was required. These IFs were most frequently found in the lungs (*n* = 5), heart (*n* = 5) and liver (*n* = 4) (Fig. 2 and Table 2). The lung IFs of three women were classified as moderate and two as major. Follow-up information was available for 1/2 women with a major lung IF. The combination of multiple pulmonary consolidations and mediastinal lymphadenopathy was suspect for sarcoidosis. The sarcoidosis diagnosis was confirmed by a follow-up chest CT scan and a bronchoalveolar lavage. Follow-up information was available for 3/5 women with a moderate heart IF. A cardiologist was consulted for all three women, two of whom (a small ventricular septal defect and a possible fistula between the left auricle and pulmonary artery) did not require further diagnostics or follow-up. The third woman with a dilated thoracic aorta underwent echocardiography and will undergo regular follow-up imaging. Follow-up information was available for 2/4 women with a moderate liver IF. The first woman underwent an ultrasound in which the liver IF was not visible. In the second woman a hypervascular lesion was found on CT and during follow-up magnetic resonance imaging (MRI) it was interpreted as liver adenoma without progression for 4 years follow-up. In 9/97 (9.3%) women, a minor IF was detected, the most frequently found IFs were cysts in the liver and a diaphragmatic hernia. More details on the minor IFs detected in women with POI are presented in Fig. 2.

#### Women with a history of PE

In the PE group using the small FOV, 23/288 (8.0%) women had one moderate/major IF, two (0.7%) women had two or more moderate/major IFs and in 23/288 (8.0%) follow-up was required. These IFs were most frequently found in the lungs (*n* = 6), heart (*n*  = 8) and liver (*n* = 7) (Fig. 2 and Table 2). 5/6 lung IFs were classified as moderate and one as major. Follow-up information about the woman with the major IF was missing, and for 3/5 women with a moderate lung IF the information was available. The first woman underwent a CT scan after 6 months to follow up three small nodules, which showed no changes and were therefore classified as irrelevant. The second woman also underwent a follow-up CT scan which concluded that the consolidation seen in the first scan was atelectasis. The third woman underwent multiple CT scans and a total body positron emission tomography (PET) CT because of growth of the lesion. Follow-up for this lesion is ongoing as there is no definite diagnosis yet. Follow-up information was available for 6/8 women with a moderate cardiac IF. Two women with a dilated aorta have regular follow-up with CT scans or echocardiography. Two women with an old myocardial infarction were referred to a cardiologist and were treaded accordingly. One woman was referred to the cardiologist because of mitral valve billowing, which did not need further investigation or treatment. One woman underwent an echocardiography because of a suspicion of an incomplete core tri-atrium sinistra, but the echocardiography did not confirm this. Follow-up information was available for 3/7 women with a moderate liver IF. In the first woman, the lesion could not be detected with an ultrasound, in the second woman the lesion was classified as a hemangioma, and in the third woman ultrasound image quality was insufficient and a multiphase CT scan was performed, classifying the lesion as a hemangioma. The CCT of one woman showed a lesion in the left breast, but the follow-up mammography did not show any suspicious lesions. A splenic artery aneurysm was found in one woman, which was confirmed by an additional abdominal CT angiography scan, but no further follow-up was necessary. Finally, the CCT of one woman revealed a mass behind the mediastinum, which was most likely a bronchogenic cyst on a follow-up MRI scan. One year later, it remained unchanged on a follow-up chest CT scan, so further follow-up is only considered necessary if she develops symptoms. In 18/282 (6.3%) women, one or more minor IFs were detected, the most frequently found IFs were cysts in the liver and a diaphragmatic hernia. More details on the minor IFs detected in women with a history of PE are presented in Fig. 2.

### Sensitivity analysis

#### Incidental findings based on double versus single reading

Sixty (21.1%) random CCTs from the UMC Utrecht were additionally assessed for IFs by a cardiovascular imaging fellow (qualified radiologist) in this center, to investigate the potential added value of double reading. This resulted in the detection of one additional liver cyst in a woman with PCOS, which was classified as a minor IF (Fig. 2). Based on this finding we concluded that single reading was sufficient.

#### Incidental findings based on small FOV versus full FOV

In a smaller subgroup, we analyzed all CCTs with an IF in Erasmus MC (*n* = 78) in a smaller FOV (Fig. 2). In 60/78 (76.9%) women with one or more IF(s) in the full FOV, the IF was still visible in the small FOV, although some were only partially depicted (Table 3); 39/61 (63.9%) of the moderate/major IFs in the full FOV were still visible in the small FOV. In the full FOV 46/78 (59.0%), women required follow-up and in 7/46 (15.2%) women clinically relevant finding were detected. In the small FOV 30/78 (38.5%) women required follow-up and in 6/30 (20.0%) clinically relevant findings were detected. Looking at the total number of IFs, 7/101 (6.9%) IFs were clinically relevant in the full FOV and 6/72 (8.3%) were clinically relevant in the small FOV. The clinically relevant finding that would be missed in the small FOV was a 7-mm lung nodule. For the follow-up of this nodule, multiple CT scans and a PET CT scan were performed and it was classified as an infectious nodule as it decreased in size by the use of antibiotics. Based on the differences in numbers of IFs in the different FOVs (Fig. 2), we decided to present the IFs in the results that were only visible in the small FOV for both centers.

**Table 3:** Comparison of incidental findings on full field of view (FOV) versus small FOV

	Full FOV-EMC (*n* = 78)	Small FOV-EMC (*n* = 78)
Women with IFs	78 (100.0)	60 (76.9)
Women with 1 IF	57 (73.1)	49 (62.8)
Women with 2 or more IFs	21 (26.9)	11 (14.1)
Number of IFs	101 (100.0)	72 (71.3*)
Minor IFs	40 (39.6)	33 (82.5*)
Moderate IFs	61 (60.4)	39 (63.9^1^)
Major IFs	–	–
Follow-up required^a^	46 (59.0)	30 (38.5)
Clinically relevant Findings^b^	7 (15.2**)	6 (20.0**)

The 78 CCTs of women with a detected incidental finding (IF) on full field of view (FOV) in the Erasmus Medical Center (EMC) were analyzed again in the small FOV (180 mm) reconstruction. Values are observed data in numbers (%)

^a^Follow-up required = the number of women for whom follow-up of the incidental finding was required

^b^Clinically relevant findings = incidental findings which required further treatment

*Percentage of number IFs in small FOV compared to full FOV

**Percentage of follow-up required

## Discussion

CCT is an established tool for the evaluation of CVD, which may also depict incidental cardiac and extracardiac findings. The aim of this cross-sectional study was to identify the spectrum and the prevalence of IFs on CCT. The overall prevalence of IFs on CCT in the total population was 19.8%, 11.1% were considered moderate/major IFs, and 9.9% required follow-up.

### Interpretation of main findings

Results from previous studies report a wide range in prevalence of IFs, depending on the patient population and type of medical imaging. A systematic review reported a median extracardiac IF occurrence on CCT of 45% (range 7–100%) [19]. The most common IFs were lung nodules or masses, lung parenchymal changes, lymphadenopathy, emphysema and liver nodules or cysts. Our population is not completely comparable to the studies in the systematic review, with a lower prevalence but a comparable nature of IFs. We included a selected subgroup of women with reproductive disorders and the number of IFs may be different in other subgroups, for example in women with a history of gestational diabetes.

Most important are the IFs that require further investigations and medical intervention. In our study, follow-up was required in 9.9% of all women, and 32.1% of the locally performed follow-up yielded a clinically relevant finding. In the systematic review of Kay et al. (2019), they found a median prevalence of only potentially clinically significant extracardiac IFs of 17% (range 1–67%) [19]. Although most IFs are benign, their discovery often causes a cascade of testing that is costly and exposes patients to additional and potentially unnecessary radiation. This may cause morbidity from adverse effects of the tests or treatments. However, for the 9 women in our study it was valuable that the study CCTA reveal their clinically relevant finding. Interestingly, depending on the pretest probability of the disease, IFs could be found more frequently than the diagnostic entities for which an imaging technique is primarily targeted [20]. In our study, in which CCTs were performed to evaluate the presence of relevant CAD in women with selected reproductive disorders, the prevalence was only 3.1–6.7%. This is a considerably lower prevalence than the above mentioned 9.9% of women that required follow-up.

A factor influencing the prevalence of IFs on CT is the FOV reconstruction. In the case of CCT imaging, coronary assessment is most often performed in a small FOV. A full FOV can be used for evaluation of non-cardiac structures but can also cause an increased prevalence of IFs. This has initiated a debate as to whether the imaging FOV should be deliberately restricted to avoid detection of non-cardiac abnormalities [14]. We investigated the impact of these different FOVs and found a higher prevalence of IFs in the full FOV (50.2%) compared to the small FOV (35.8%). Furthermore, with the full FOV 46/78 (59.0%) women required additional appointments or diagnostics for follow-up of their IF, and in the small FOV only 30/78 (38.5%) women would be referred for further follow-up. In terms of relevant findings, one infectious lung nodule was detected with the full FOV, which would have been missed in the small FOV. To summarize, our findings indicate that the benefits of using a full FOV do not outweigh the patient burden caused by the extra follow-up. 

Among radiologists, there is an ongoing debate about reporting minor IFs that have no clinical consequence. Usually there are local agreements between clinicians and radiologists whether a radiologist reports all findings, and the clinician must interpret the importance, or whether the radiologist shares responsibility for interpreting the finding’s importance and only reports IFs that have clinical consequence [21]. Reporting only potentially clinically relevant IFs could reduce anxiety and extra costs for downstream diagnostic testing.

### Methodological considerations

This is the first study that provides insight into the prevalence and nature of IFs in women with PCOS, POI or a history of PE. An important strength of this study is the size and characteristics of the population. We provided an overview of the required follow-up and discussed some important considerations about the technical execution of the CCT, such as FOV and double reading.

A limitation of this study was the difference in local protocols between the two centers, leading to differences in single and double reading and in FOV reconstruction. Both may cause different detection rates of IFs. However, we demonstrated that the impact of double reading was very low. It also gave us the opportunity to assess the impact of using different FOVs. A smaller FOV leads to a lower prevalence of IFs and thus required less follow-up, while only one clinically relevant finding would be missed.

There was an uneven distribution of women with PCOS, POI or a history of PE between the two centers. However, the purpose of the study was not to compare the groups, as we have no reason to assume that detecting IFs in either group is more likely. Comparing our total group with healthy women with the same age may give a better overview of the difference in IFs between women with or without these reproductive disorders. To the best of our knowledge, a comparable control group was not available in the literature.

Not all follow-up information was available due to different follow-up pathways. However, with the available information we can conclude that detecting moderate/major IFs has a considerable consequence in most participants.

## Conclusion

The rapid acceptance of CT screening for cardiovascular disease increases the risk of detecting incidental findings. It is important to take the harm and benefits into account and counsel participants adequate about CCTA. On one hand, it can reveal clinically relevant findings, and on the other hand, it can cause anxiety and may generate extra costs for patients. This cross-sectional study shows that the prevalence of one or more IFs in middle-aged women with a reproductive disorder (PCOS, POI or a history of PE) is between 15 and 32%. Detected IFs required follow-up in 48/486 (9.9%) women, leading to 9/486 (1.9%) clinically relevant findings. A small FOV centered around the heart resulted in a lower prevalence of IFs and required less follow-up, while only one clinically relevant finding would have been missed.

## Data Availability

The data underlying this article will be shared on reasonable request to the corresponding author.

## Abbreviations

BMI: Body mass index
CAC: Coronary artery calcium
CAD: Coronary artery disease
CCT: Coronary computed tomography
CCTA: Contrast-enhanced computed tomography angiography
CREw-IMAGO: Cardiovascular Risk Profile-Imaging and Gender-Specific Disorders
CVD: Cardiovascular diseases
FOV: Field of view
HDP: Hypertensive pregnancy disorders
HRT: Hormone replacement therapy
IF: Incidental finding
MRI: Magnetic resonance imaging
PCOS: Polycystic ovary syndrome
PE: Preeclampsia
PET: Positron emission tomography
POI: Premature ovarian insufficiency
